# The experiences of people bereaved by suicide regarding the press reporting of the death: qualitative study

**DOI:** 10.1186/s12889-020-8211-1

**Published:** 2020-02-20

**Authors:** Philip Gregory, Fiona Stevenson, Michael King, David Osborn, Alexandra Pitman

**Affiliations:** 1 UCL Division of Psychiatry, University College London, 6th floor, Maple House, 149 Tottenham Court Road, London, W1T 7NF UK; 2 Barnet Enfield & Haringey Mental Health NHS Trust, London, UK; 3 Camden and Islington NHS Foundation Trust, London, UK; 4 UCL Department of Primary Care and Population Health, University College London, London, UK

**Keywords:** Suicide prevention, Media reporting, Suicide bereavement, Qualitative, Thematic analysis

## Abstract

**Background:**

Media guidelines on suicide reporting of suicide have two purposes: to prevent further suicides, and to minimise distress to the bereaved, who are themselves at increased risk of suicide. We aimed to describe the subjective experiences of people bereaved by suicide regarding media reporting of the suicide of their friend or relative.

**Methods:**

We conducted a cross-sectional study of staff and students aged 18–40 at 37 United Kingdom higher educational institutions in 2010 to recruit adults who had experienced bereavement by the suicide of a close contact. We analysed free-text responses to a question probing experiences of the press after the suicide, using thematic analysis to identify key themes.

**Results:**

We analysed responses from 140 eligible respondents, and identified 3 main themes: value placed on respecting the privacy or wishes of the bereaved; respect accorded to the deceased; and the role of the press in promoting suicide prevention messages. Many respondents described negative experiences of the press, with sub-themes capturing distressing experiences relating to perceptions of journalists’ intrusive behaviour, failure to consult appropriately with the bereaved, journalists releasing private information, negatively misrepresenting the deceased, and breaching the anonymity of the deceased or bereaved. We identified considerable variation in people’s views over acceptable levels of detail reported in the press, and in some cases objections were in relation to journalists following media guidelines. These divergent views illustrate the tensions between the twin purposes of media guidelines: to prevent further suicides, and to protect the bereaved.

**Conclusions:**

The findings from our British sample provide journalists with personal perspectives from bereaved relatives on the impact of media intrusion, speculation, and misrepresentation, and an insight into disparate views on the nature of information relatives feel comfortable disclosing. These findings suggest a need for journalists’ training to include exposure to such views, to heighten awareness of potentially distressing effects and the nuances of bereaved people’s preferences. This should aim to encourage journalists to consult with bereaved relatives more sensitively, whilst also remaining mindful of media guidelines on the reporting of suicide.

## Introduction

News journalists are sometimes expected to report on suicides, and whilst their first responsibility is to report the facts, it can be difficult to know how convey these in a manner that does not cause distress to bereaved relatives. The additional and linked challenge that journalists face is the growing body of evidence [1–3] that irresponsible reporting of suicide is associated with increases in suicides at the population level. Younger people and the elderly are thought to be most susceptible to the emulative influence of widely-publicised suicides, also termed the Werther effect [2]. Mechanisms remain unclear, but are likely to involve explanations such as identification, social modelling, and cognitive availability of methods [4]. Concern about these two problems has led many countries to include in their suicide prevention strategies the recommendation that media agencies should be supported in delivering sensitive approaches to suicide and suicidal behavior [5, 6]. These emphasise that media coverage of suicide offers opportunities to prevent further suicides if journalists follow best practice.

National [7, 8] and international [9] media guidelines advise journalists to avoid glorifying the death or detailing the method, and to include information on sources of support for readers affected by the issue of suicide. Additionally, Samaritans guidelines advise that reporters “*must guard against intrusion into the grief and shock of the bereaved while considering industry regulation and codes of practice*”. World Health Organization (WHO) guidelines [9], and the British editors’ code of conduct [8], advise applying caution when interviewing bereaved family or friends, avoiding intrusion into grief or shock, and being sensitive about details published. Mitigating any distress caused to those grieving is critical because suicide bereavement increases the risk of suicide and psychiatric illness [10, 11]. Registry-based studies provide clear evidence that the offspring [12], parents [13], and spouses [14, 15] of those who die by suicide are subsequently at risk of suicide. Friends are also at risk of suicide attempts [16], indicating wide effects on social networks. Adding to the burden of grief through insensitive reporting may further traumatize the bereaved, as evidenced in previous qualitative accounts [17]. Such work illustrates the potential for tensions between what media guidelines recommend and the preferences of the bereaved, particularly in relation to providing a public memorial of the deceased or describing the method used as a means of highlighting prevention opportunities [17].

Studies in the United States [18], China [19, 20], India [21], Sri Lanka [22], Britain [23–26] and Ireland [24, 27], suggest that newspaper journalists’ adherence to media guidelines on reporting suicide is generally poor, and similarly for online news reporting [20, 28]. Two recent analyses of media content reporting suicidality found that 87% of British content [28] and 99% of Irish content [27] failed to comply with at least one of the Samaritans' guidelines. Factors implicated in the low adherence of journalists to such guidelines include a lack of involvement in their development [25], scepticism about the damaging effects of suicide reporting [29], and a lack of awareness of guidelines. Educating journalists about the emotional impact of such reporting on bereaved individuals, and also the wider public health evidence for damaging effects of insensitive reporting, may help reinforce the importance of adhering to guidelines. There is a need for audience reception studies describing the impact of different styles of reporting of suicide on specific high-risk populations, particularly those people bereaved by the suicide. This would both improve our understanding of support needs after a suicide loss, but also support collaborative working with journalists to reduce any identified negative effects. Our objective was to elicit the views of a population-based sample of young adults bereaved by suicide on the media’s response to the suicide of their friend or relative. By using a national online survey to elicit qualitative accounts, and an inductive approach to explore whether these experiences were positive or negative, we aimed to illustrate the impact of the media on the bereaved after a suicide.

## Methods

### Study design and participants

We invited all adults aged 18-40 years who were working or studying at United Kingdom (UK) higher education institutions (HEIs) to participate in a closed, online study about sudden bereavement: the UCL (University College London) Bereavement Study. Recruitment for this survey has been described previously [16]. Briefly, we used the all-staff/all-student email systems of 37 UK colleges and universities (of the total of 164 HEIs at that time) to send individual emails to a large and varied but defined sample of young adults. This was judged to be the best means of accessing hard-to-reach groups, whilst avoiding the biases associated with recruiting a help-seeking sample [16]. The email invited a sampling frame of 659,572 staff and students to take part in a survey of “*the impact of sudden bereavement on young adults*”. There was no accurate way of measuring response rate as the denominator of bereaved people in this sample was not ascertainable using routine data or survey methods.

Inclusion criteria were as follows: people aged 18-40 years who, since the age of ten years, had experienced sudden bereavement of a close friend or relative. The 18-40 age range was chosen to reflect an under-researched group of great interest in suicide prevention policy. Early childhood bereavements were excluded to minimise recall bias and restrict our focus to adult cognitive processing of life events, using the age threshold for criminal responsibility in England and Wales. A close contact was defined as “*a relative or friend who mattered to you, and from whom you were able to obtain support, either emotional or practical*”. Sudden bereavement was operationalised as “*a death that could not have been predicted at that time and which occurred suddenly or within a matter of days*”. The cause of death was classified by responses to the question: “*Since you were aged 10 have you experienced a sudden bereavement of someone close to you due to any of the following: a) sudden natural death (eg. cardiac arrest, epileptic seizure, stroke); b) sudden un-natural death (eg. road crash, murder or manslaughter, work accident); c) suicide?*” Cause was therefore defined subjectively by the respondent, and not by coroner’s verdict or death certificate, as we were primarily interested in respondents’ perceptions of the cause. For people bereaved by more than one suicide, respondents were asked to relate their responses to the person who they had felt closest to.

### Procedures

The on-line questionnaire [16] was designed by AP, FS, DO, and MK to answer a range of research questions using quantitative and qualitative approaches. This was in consultation with a group of young bereaved adults and bereavement counsellors, who suggested which domains to cover and the appropriate wording of questions. Part 1 contained 119 fixed-response questions eliciting quantitative data on socio-demographic and clinical characteristics. Part 2 contained 20 open questions to elicit free text qualitative data on research questions about specific dimensions of the impact of bereavement. Survey questions were intended to be non-leading and neutral, so as to avoid assuming only negative associations of bereavement. The questionnaire was piloted as an open survey on the websites of the four national voluntary sector organisations (Samaritans, Cruse Bereavement Care, Survivors of Bereavement by Suicide, and Widowed by Suicide). We used responses to make changes to the wording of specific questions.

One open question probed respondents’ experience of the press reporting of the death, and was worded: “*Please describe any positive or negative experiences you may have had after the death in relation to the following: police force; funeral directors; coroner's office; healthcare staff; press reporting on the death*”. There was no upper word limit and respondents were invited to give as much or little detail as they wished, or to skip the question if it did not apply.

The participant information sheet indicated that the study was being conducted by a research team at UCL, including research psychiatrists (AP, DO, MK) and a medical sociologist (FS). It explained that results would be analysed and compiled into a publically available report, and that no individual respondent would be identifiable from this information.

### Ethical approval

All participants provided online informed consent. The study protocol was approved by the UCL Research Ethics Committee in 2010 (reference: 1975/002).

### Analytic approach

We imported online responses to the question on press experiences into Microsoft Excel, which allowed us to organize, review, and code large volumes of relatively brief textual data. We restricted our analysis to responses from people bereaved by suicide, and who specifically mentioned the press in their response to this question. We used two stages of analysis to organize this number of datalines. Initially we organised responses into a basic content-based classification of positive and negative experiences of press reporting, based on the subjective accounts of the bereaved, before progressing to a more fine-grained thematic analysis [4]. Initially two researchers (PG & AP) coded all responses independently, having familiarised themselves with the data. Having compared codes to assess inter-rater reliability, and calculated Cohen’s kappa, raters agreed on an initial basic classification of responses based on two dimensions: whether the experience had been positive or negative. We then revised this, based on degree of elaboration, to create a six-part classification as follows: brief positive responses, brief negative responses, extended positive responses, extended negative responses, extended responses capturing both positive and negative experiences, and uninterpretable responses.

We then excluded all uninterpretable responses, and two researchers (PG & AP) proceeded to a deeper exploration of the meaning of participants’ experiences, by independently coding all extended responses within this classification to identify key themes. Having compared coding frameworks to review consistency between coders as a check on robustness, and agreed an initial coding framework, PG then recoded the full dataset, building up a framework of new codes, sub-codes, and collapsed codes in collaboration with AP. Both then reviewed sub-codes against higher-order themes to validate the coding framework and ensure conceptual coherence. Regular discussion meetings within the research team encouraged reflexivity and enhanced validity by providing opportunities to question and refine our interpretations and analytic processes, and to provide further validation of the conceptual coherence of thematic codes. Data were then reviewed against higher-order themes as a final validation of the conceptual meaning of the analysis.

We followed COREQ (consolidated criteria for reporting qualitative research) guidelines on the reporting of qualitative research [30], providing quotes as typewritten in online responses, corrected only for minor spelling errors.

## Results

### Response

**Fig. 1:**
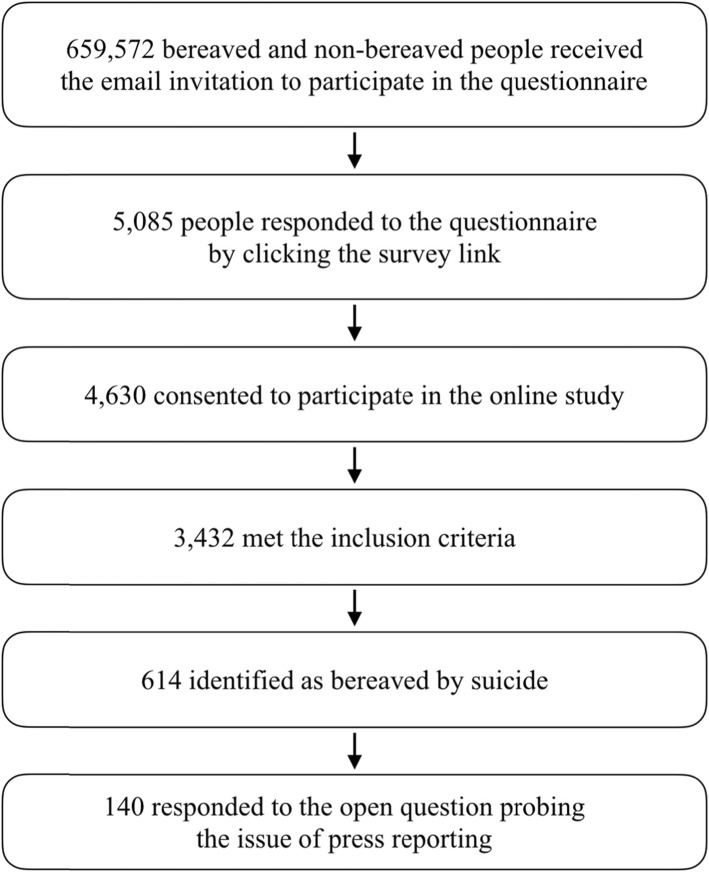
Participant flow

Of the estimated 659,572 people receiving the email invitation, 5,085 people responded to the questionnaire by clicking on the survey link, and 4,630 (91%) consented to participate in the online study (see Fig. 1). Of the 3,432 participants meeting inclusion criteria, a total of 614 identified as having been bereaved by suicide, of whom 351 responded to the question capturing experiences of the police force, funeral directors, coroner’s office, healthcare staff, or the press. Of those 351 responses, 140 specifically mentioned the press in their responses. We therefore analysed free text responses for a sample of 140 respondents.

### Characteristics of sample

Our sample (Table 1) was predominantly female (83%), students (88%), of white ethnicity (95%), educated to degree level and above (78%), and of higher socio-economic status (59%). Respondents had a median age of 23 years (inter-quartile range [IQR]=8; mean=25.0; standard deviation [SD]=6.0). The median age at bereavement was 19 years (IQR=6), with 36% (51/140) having experienced the bereavement below the age of 18 years. Mean time elapsed since bereavement was 5.5 years (SD=5.5). In 77% of cases the deceased had been male. The mean age of the deceased was 33.0 years (SD=16.7 years; median= 27.5; IQR=26.5). Equal proportions reported the suicide of a family member (49%) *versus* that of a non-relative (50%). Kinship to the deceased was most commonly a friend or colleague (31%), followed by father (19%), brother (9%), partner (8%), cousin (6%), uncle/aunt (6%), and mother (4%).

**Table 1:** Socio-demographic characteristics of study participants (*n*=140)

Characteristics	number (%)
Characteristics of the bereaved	
Age	
18-21	52 (37%)
22-40	88 (63%)
Gender	
Male	24 (17%)
Female	116 (83%)
Marital status	
Co-habiting	33 (24%)
Divorced	1 (1%)
Married/civil union	20 (14%)
Single	86 (61%)
Socio-economic status ^a^	
SE status 1-2	83 (59%)
SE status 3-7 and 9	54 (39%)
Ethnicity	
White	133 (95%)
Non-white	7 (5%)
Student/staff status	
Student	123 (88%)
Staff	13 (9%)
Both staff and student	4 (3%)
Relationship to deceased ^b^	
Blood relative	70 (50%)
Not a blood relative	69 (49%)
Characteristics of the deceased	
Age of deceased	
Under 18	24 (17%)
Over 18	116 (83%)
Gender of deceased	
Male	108 (77%)
Female	32 (23%)
Time since death	
Less than 2 years	34 (24%)
2 years and over	106 (76%)

^a^ Socio-economic status was derived from a question about own occupation (for university staff) or parental occupation (for students), using the five categories from UK Office for National Statistics (ONS)

^b^ For people bereaved by more than one suicide, respondents were asked to relate their responses to the person who they had felt closest to.

### Basic response characteristics

Our initial basic classification of 140 responses identified minimally overlapping categories of 29/140 people (21%) with positive experiences, 92/140 (66%) with negative experiences, and 17/140 (12%) with neutral experiences. Inter-rater reliability was high, with a kappa value of 0.9370. Based on the degree of elaboration of responses, we developed a more descriptive six-part categorization : brief positive responses (n=10; 7% eg “*press were excellent”*); brief negative responses (n=11; 8% eg “*the press were cruel”; “the press made it worse”)*; elaborated positive responses (n=11; 8%); elaborated negative responses (n=73; 52%); elaborated responses capturing positive and negative experiences (n=8; 6%); uninterpretable responses (n=10; 7%; e.g. “*Deaths by suicide are generally not reported upon by the media*.”; “*I have seen press clippings. It was the first time that I had found out the type of gun*.”*)*; and neutral responses (n=17; 12% *“neither positive nor negative experiences”*). Neutral responses tended to convey recalling little about press coverage (e.g. “*they did their job. I don’t really remember much about them.”)* or reflecting that this may have been hidden from them (eg. “*was very young and was not aware of much of their involvement.*”; “*I know the press reported on the death. I want to know what they said as it was always hidden from me as a child.*”). Responses related primarily to newspaper journalism, but also mentioned TV coverage.

### Themes identified

Our more fine-grained thematic analysis of responses identified three key themes representing bereaved people’s experiences of the press reporting of the suicide (Table 2): 1) Value placed on respecting the privacy or wishes of the bereaved (sub-themes a) intrusive behaviour of journalists; b) importance of consultation with the bereaved; c) press taking control over information released; d) divergent views over level of acceptable detail); 2) Respect accorded to the deceased (sub-themes a) balancing a focus on the deceased’s life achievements versus their death; b) negative portrayal of the deceased’s character); and 3) The role of the press in promoting suicide prevention messages. These are described below, illustrated with quotes. Responses from each participant were coded under up to three themes. In relation to socio-demographic factors, themes varied little by gender, age, or kinship.

**Table 2:** Table describing main themes and sub-themes

No	Theme	Subtheme
1	Value placed on respecting the privacy or wishes of the bereaved	
	1A	intrusive behaviour of journalists
	1B	importance of consultation with the bereaved
	1C	press taking control over information released
	1D	divergent views over level of acceptable detail
2	Respect accorded to the deceased	
	2A	balancing a focus on the deceased’s life achievements versus their death
	2B	negative portrayal of the deceased’s character
3	The role of the press in promoting suicide prevention messages	

#### Value placed on respecting the privacy or wishes of the bereaved

In the first theme, many respondents commented on the value they placed on journalists respecting their privacy or their wishes about how the death should be reported. However, these accounts were primarily based on their negative experiences of press intrusion and of their wishes not being respected.

##### Intrusive behaviour of journalists

It was common for respondents to report that they had found journalists’ behaviour inappropriate due to their intrusive approach in pursuing the story. The methods journalists used to elicit information about the death left the bereaved feeling hounded, particularly where journalists used deceitful tactics.

*“Press were entirely negative as I remember it. They would come to the house asking questions, prying.” (male in his 20s, bereaved 11 years previously by the suicide of his sister)*

*“The press hounded her parents for a statement to the point where they printed one off and shoved it out the letter box to them. They followed her friends around even though they knew we didn’t want to talk to them.” (female in her 20s, bereaved 10 years previously by the suicide of a close friend)*

A few respondents described being surprised or saddened to discover journalists at the coroner’s inquest or the funeral, experiencing discomfort or distress at this intrusion. Such events were felt to be particularly important times at which the privacy of the bereaved should be respected.

*“I was alarmed and angered by the presence of journalists at the inquest who appeared like vultures at the back of the room taking notes. I was further angered by their inaccurate reports which appeared in the local papers.” (female in her 30s, bereaved 7 years previously by the suicide of her step-grandfather)*

*“The press reporting on the death were nothing short of a nightmare, turning up with TV cameras outside the house. Constantly knocking on the door wanting information and photographs, and finally appearing and reporting on the funeral.” (female in her 20s, bereaved 1 year previously by the suicide of her mother)*

The experience of seeing the story in the press was also described as intrusive, particularly when it appeared repeatedly, was inaccurate, or where the story was reactivated after the inquest.

*“ … I avoided the papers for at least a week. And suddenly a few months later when the coroner's report was done, it was back on the front page. It's naive stories told by people who didn't know him at all … ” (male in his late teens, bereaved 1 year previously by the suicide of a close friend)*

*“I hated it appearing in the local paper, over numerous weeks.” (female in her 30s, bereaved 5 years previously by the suicide of her step-brother)*

##### Importance of consultation with the bereaved

Some participants commented on how upset they had been over not being consulted over the portrayal of the death in press reports. Journalists’ pursuit of a good story seemed to them to outweigh a need to consult the family or consider their feelings.

“The press were b*******, pure and simple. They ran with it for weeks in the local paper, without ever consulting the family or appreciating their loss. They were all about the (statutory service’s) failings, without remembering the death.” (male in his 20s, bereaved 5 months previously by the suicide of his cousin)

*“the press were just joyful of a story.” (female in her 30s, bereaved 3 months previously by the suicide of her brother)*

This lack of consultation also led to the bereaved feeling resentful that journalists had not sought their consent for disclosure of personal information, apparently sourced from social media or police statements and then propagated widely. This broadcasting of information left many feeling exposed and judged by people reading the articles, and angry at the violation of their right to privacy over personal matters.

*“I did not have a lot of contact with (the press), however I was mentioned in a local news article, which was written without our consent, and used personal information about me which I think they found on Facebook.” (female in her 30s, bereaved 3 years previously by the suicide of her brother)*

A small number of data outliers revealed contradictory views regarding consultation, as some respondents were disappointed that the press had not paid due attention to the death. These exceptional data implied that, with the appropriate collaboration, an article about the death could have offered a fitting tribute. Such competing views highlighted how important it was for journalists to clarify specific preferences.

*“I felt angry that his death wasn't given more coverage.” (female in her 20s, bereaved 5 years previously by the suicide of a close friend)*

*“It wasn't even reported in the local paper and this made me feel sad as it was like the person didn't mean anything to anyone.” (female in her 30s, bereaved 17 years previously by the suicide of a close friend)*

The very rare positive examples of press consultation reinforced the value placed on consultation, with the bereaved willing to provide photographs or an account of the death providing that they retained some control over the details presented. This contrasts with some of the experiences presented later under sub-theme 1D (Divergent views over level of acceptable detail), describing objections to the level of detail provided by journalists who had not consulted appropriately.

*"They were sensitive with what they wrote and asked for our comments and they put a nice photo in. It went on the front page of the local paper.” (female in her 30s, bereaved 6 years previously by the suicide of her brother-in-law)*

Together the data within this theme suggested that feeling positively involved in the press coverage might be an important part of the processing of the loss.

##### Press taking control over information released

Some participants described feeling a loss of control over the type of information (or level or detail) included in press reports. It worried them that some people in the deceased’s social circle might have the news broken to them through hearing about it in the media, before the next-of-kin had the chance to tell them in person.

*“There was one report in a newspaper from the area it happened. I found it on the internet. I didn't tell my best friend because I didn't want to upset her more but in the end her and her sister discovered it anyway … ” (female in her 20s, bereaved 4 months previously by the suicide of a close friend)*

This was a particular issue where family members were hoping to hide the cause of death from certain individuals, particularly children. The negative impacts on children were apparent where their peers learnt about the death from press reports, and in some cases teased them about it.

*“Press reported the details in our local newspaper following the court case. It was awful, I didn't want people knowing our business. Again I felt that other people wouldn't understand and that they would judge grandad.” (female in her 30s, bereaved 25 years previously by the suicide of her grandfather)*

The stress of knowing that this information was in the public domain was difficult, perpetuating anxiety that vulnerable others, unaware of the cause of death, might find out this hidden information.

*“My grandmother was very ill around this time so we did not explain to her the cause of death. She could not cope with her child dying before her. We had constant worry about her reading something in the paper or someone saying something to her. To this day she still does not know, therefore we talk around the subject, saying he died of a massive heart attack.” (female in her late teens, bereaved 8 months previously by the suicide of her uncle)*

##### Divergent views over level of acceptable detail

A specific issue that many respondents commented on was their reaction to the level of detail provided in the article, with a divergent range of opinions as to what was acceptable. These comments related to naming of the deceased or their relatives, specifying suicide as the cause of death, publishing the deceased’s photograph, and providing graphic details about the suicide method. Where the press did not reveal the deceased’s identity this was often a relief. However, as indicated in sub-themes 2A (Balancing a focus on the deceased’s life achievements over their death) and 2B (Negative portrayal of the deceased’s character), where the deceased was identified in relation to their contribution to society this was felt to be an acceptable press response.

*“Unfortunately the press did find out about his death and there was quite a bit of negative press about it but luckily no names were revealed. This helped a lot for myself emotionally.” (female in her late teens, bereaved 1 year previously by the suicide of her partner)*

Views differed on whether reports should have stated publicly that the death was a suicide. Respondents either valued the journalist’s avoidance of the word suicide, or resented them for misleading readers over the real cause, even given the assumption that the journalist’s intention had been to protect the family. Both positions arose from situations in which there had been no apparent consultation with the family, suggesting that the journalist had no means of checking whether their reporting was in line with what that family might deem acceptable. Again, as with sub-themes 1B (Importance of consultation with the bereaved) and 2B (Negative portrayal of the deceased’s character), this stressed the importance of seeking the views of the bereaved.

*“the press didn’t report suicide in the papers; which I thought was really tactful, they only ever said 'sudden death’.” (female in her late teens, bereaved 3 years previously by the suicide of a close friend)*

*“The press stated her death was an accident and lied about it. I didn't think they needed to lie about it.” (female in her 20s, bereaved 11 years previously by the suicide of a close friend)*

The use of photographs of the deceased was a contentious issue. Seeing a photo published was often a shock for which family or friends were unprepared. Using an unflattering, poor quality, or incorrect photo was also upsetting for the lack of respect it seemed to accord to the deceased or their family. Again, the lack of consultation over the use or choice of images was highlighted.

*“and one time it was again in the paper with a photo, which I was unprepared for seeing.” (female in her 30s, bereaved 5 years previously by the suicide of her step-brother)*

*“Press were terrible, one particular paper published a photo of the wrong person with the story which shocked me at their carelessness.” (female in her late teens, bereaved 3 years previously by the suicide of her ex-partner)*

Respondents expressed unanimous disapproval of providing details of the method of suicide on the basis that this was upsetting for family members to see, and also felt to be both unnecessary and disrespectful.

*“there was a small column in the paper about how a man was found hanged in his flat. We removed the page from the paper so not to upset my grieving aunt.” (female in her late teens, bereaved 11 months previously by the suicide of her uncle)*

*“One particular newspaper reported the incident in very disrespectful and graphic terms which my friend complained about on my behalf.” (female in her 30s, bereaved 15 years previously by the suicide of her partner)*

The range of views under this theme revealed the extent to which people’s limits of acceptability differed over how they wished the death to be reported. Those who were happy for personal messages from wreaths to be published lay in contrast to those who might regard this as intrusive. Again this sub-theme highlighted the importance of consultation in checking personal values and preferences.

*“I was angry with the press, because they reported her death in a manner I did not feel necessary and revealed too many details.” (female in her 20s, bereaved 2 years previously by the suicide of her aunt by marriage)*

*“ … and they posted the messages we left on flowers in the local paper which I thought was a really nice thing to do.” (female in her 20s, bereaved 10 years previously by the suicide of a close friend)*

#### Respect accorded to the deceased

The second main theme captured the importance that the bereaved placed on media reports paying respect to the deceased. Again, this was primarily based on the negative experiences of those who had perceived journalists showing a lack of respect for their deceased relative or friend.

##### Balancing a focus on the deceased’s life achievements *versus* their death

One of the ways that press coverage was perceived to show respect for the deceased was in describing their life achievements and not reducing them to the stark details of their demise. Reports focusing solely on their death were seen as undermining of that individual, depersonalizing them in pursuit of a morbid story. This was particularly resented where articles included conjecture around the triggers for their suicide.

*“Press focused too much on how they died and not about the achievements during their lifetime.” (female in her late teens, bereaved 9 years previously by the suicide of her uncle)*

*“press report was a bit unpersonal and I think it should of explained what type of person, how many children etc that he had” (female in her 20s, bereaved 5 years previously by the suicide of her father)*

Some perceived an inappropriate focus on distracting details, such as the deceased having a famous relative or acquaintance, detracting from their own life’s achievements. These quotes are not presented to avoid identifying individuals. In perceiving the inclusion of these details as a ‘selling point’ for press reports, there was a sense that respect for the dead had been sacrificed in pursuit of a headline that would grab the public’s attention. A solitary account of respect being accorded to the deceased related to a respondent describing the press response to the death of her father, who had been well-known in his professional field. In this case the press coverage was gratifying for her in honoring her father’s contribution to society.

##### Negative portrayal of the deceased’s character

One particularly upsetting way in which the press showed disrespect for the deceased was in portraying them in negative terms. This was either through reporting inaccurate or misleading information, or stereotyping their character based on superficial details of their profession, relationships, or difficulties.

*“Negative experience of the press who made my brother out to be a nasty person when really he was just a tormented soul who had a very gentle nature.” (female in her 20s, bereaved 3 years previously by the suicide of her brother)*

*“The press made him sound like a different person, they tried to blame music and art for his suicidal thoughts.” (female in her 20s, bereaved 3 years previously by the suicide of a close friend)*

This sub-theme overlapped with sub-theme 1B (importance of consultation with the bereaved) in highlighting the use of speculation or the accounts of peripheral contacts rather than consulting sensitively with those closest to the deceased. In practice this may have arisen where journalists were not able to gain interviews with next-of-kin. However, a reliance on peripheral contacts was reported to result in inaccurate characterisations based on impersonal accounts from detached and disinterested people. It thus overlapped with the sub-theme 2A above in misrepresenting the deceased’s life achievements.

*“Press reporting on the death, particularly with respect to the local obituary, were extremely upsetting for close family and friends! i.e. people who barely knew him (though used him for drugs etc) left condolences that indirectly indicated that his relationship with my sister was the cause of his death.” (male in his 20s, bereaved 2 years previously by the suicide of his sister’s partner)*

*“The press and many friends that met him within the last year or two focussed on this barman persona. It was upsetting to myself and others as it reduced him to a label and felt impersonal and pointless.” (male in his 20s, bereaved 9 months previously by the suicide of a close friend)*

#### The role of the press in promoting suicide prevention messages

In the third theme, a few participants commented on the potentially positive value of the press in raising awareness of mental illness and educating the public about suicide. They provided instances in which this effect had been achieved either spontaneously or in consultation with the bereaved.

*“A local journalist has been very kind in reporting on the case in order to raise awareness of suicide with the help of my mother and I.” (female in her 20s, bereaved 9 years previously by the suicide of her brother)*

*“When the second twin committed suicide the press reported on it in a bid to raise awareness of suicide as the family had lost 2 daughters in 9 months” (female in her 20s, bereaved by the suicides of two cousins within the previous year)*

However, others presented examples of missed opportunities for the press to have used their influence to communicate valuable health promotion messages. Direct efforts by the bereaved to involve the press in suicide prevention were not always successful. For example, requests to convey warnings about risk factors were sometimes ignored in favour of sensational headlines.

*“The deceased was secretly a member of a pro-suicide forum where strangers encourage each other and give advice on the best ways to commit suicide. His family raised the issue in the local newspaper in an attempt to have websites like these banned, they gave a brief account of the causes behind the bipolar disorder which he suffered from, including some examples of instances of bullying. Disappointingly, the newspaper ignored the whole point of the article and gave it the headline 'Teenage suicide was victim of bullying’.” (female in her late teens, bereaved 2 years previously by the suicide of a close friend)*

This third theme revealed the preparedness of `some bereaved people to engage with the press for a greater cause, and the hope they placed in the willingness of the press to collaborate on this.

## Discussion

### Main findings

In this sample of people bereaved by suicide, we identified divergent views about the level of detail that should be included in press reports. Although most respondents described negative experiences of press coverage of the suicide, sometimes this related to inadequate details of the death. Our thematic analysis found that negative experiences related to press intrusion, involving deceitful methods of information-seeking, inaccurate representations of the person who died, and a focus on sensationalist details. Disgust and distress were common reactions to these experiences. Inquests and funerals were particularly difficult times, made harder by the presence of the press. Some described lacking a sense of control over what others found out about the suicide, due to press reporting details of the death. Factors like a lack of consultation, factual inaccuracies, and excessive detail gave the bereaved the impression that the journalists concerned had given little thought to the impact on the bereaved and their vulnerabilities. For many, journalists’ sensationalist portrayal of the death seemed to expose their goal of gaining the readership’s attention rather than accurately reflecting the facts. Reporting styles included picking out unattractive details of the person’s character or relationships, or including misleading speculation as to the triggers for the suicide. Respondents felt upset that their loved one’s death was being exploited in this way, particularly in cases of selective reporting. This was reinforced where the story was featured prominently, for example on the front page, or for a protracted period. Some respondents accepted that journalists were reporting on the story as part of their job. However, as one female respondent in her 20s commented, five years after her partner’s suicide, “*I know it’s their job, but they made everything much harder*.”

Our analysis identified conflicting views of participants over issues such as the acceptable level of detail in reports, and the appropriate balance between reporters’ attempts at consultation *versus* perceived intrusion. Preferences over revealing the cause of death ranged from irritation at concealment to a desire for vaguer terms (such as sudden death). Generally suicide-bereaved people seemed prepared to engage with the press to ensure that no inaccurate or misleading details were reported, and to contribute to an article honouring the life of the deceased, or where they perceived coverage as promoting suicide prevention. The time implications of this engagement may not have felt realistic to the journalists concerned. Publishing photos or information was felt to be unacceptable if obtained in underhand ways, but not if negotiated fairly. The diversity of views on these issues suggested that consultation with the bereaved, conducted in a sensitive way, is an important step in clarifying preferences. However, this also demonstrated that sometimes the views of the bereaved on appropriate media reporting are at odds with those of policymakers.

There was significant overlap in the themes identified. The importance of accuracy was highlighted in relation to the perceived intrusiveness of publishing incorrect reports, the disservice of mis-portraying the deceased, and the insult of inaccurate reporting. This was seen to show a lack of respect for the deceased and bereaved survivors, and a desire to sell papers. The importance of consultation recurred across sub-themes, often in relation to addressing the issue of inaccuracy. It was striking that strong views about privacy and press intrusion were expressed by those across the social network, and not just immediate family.

### Results in the context of other studies

Our findings of experiences of press intrusion, and of divergent views on appropriate level of detail, are consistent with those of two previous qualitative studies amongst the suicide-bereaved, one British [17] and one Australian [31]. A British interview study found that whilst bereaved relatives held an expectation of sympathetic and accurate reporting, they were sometimes keen to provide the press with details of the death or images of the deceased if this improved accuracy [17]. The authors noted that this contradicted press guidelines emphasising avoidance of details on method used or photos of the deceased. As in our study, significant distress arose from careless reporting, speculation, and inaccurate impressions of the death, but opportunities were also identified for suicide prevention messages. The Australian study noted that the bereaved found it unhelpful to engage with the media in the immediate aftermath of the loss, and sometimes found the press intrusive [31]. While identifying that some bereaved individuals were motivated to engage with journalists altruistically to help others, the authors also noted the potential conflict with journalists’ motivation to publish an arresting story [31]. Together these findings reinforce what is enshrined in the British Editors’ codebook on accuracy, privacy, harassment, and intrusion into grief [8], but also highlight the contradictions that arise in adhering to media guidelines whilst also respecting relatives’ wishes.

### Strengths and limitations

We surveyed a large but defined sample of UK-based adults bereaved by suicide using a representative method of sampling people working or studying in UK HEIs. However, whilst we elicited a wide range of views from those who responded, our method resulted in over-representation of white, highly-educated, articulate females and perhaps those voicing more extreme experiences of the press. There was little representation of men bereaved by the suicide of a male peer or relative. These response biases limit the resonance of our findings to other groups. Our initial basic classification was intended to convey the balance of positive *versus* negative experiences, albeit superficially. The question we used to elicit experiences of the press was one of a set of open questions probing aspects of bereavement, and in analysing online data from this question alone we may have missed the context provided by other responses. This question also probed experiences of four other agencies (police force; funeral directors; coroner's office; healthcare staff), whereas asking a specific question about media coverage may have provided a fuller account of press behaviour. This approach may also have been more likely to have elicited views from those with more negative experiences of the press. Our wording may have primed respondents to describe experience of the print media rather than radio, television, or online coverage. Further work would be needed to explore responses to online reporting, including Twitter. Data collection occurred nine years ago, and an exploration of more recent experiences of the bereaved would provide a more valid account of the effects of media reporting, assuming greater current awareness of media guidelines. Due to the nature of data collection we were unable to ascertain whether the media content described was published in broadsheet or tabloid newspapers, or whether the journalists concerned were staff or freelance, and this limits how we can use the findings to target journalists’ training appropriately. We were also unable to distinguish in all cases between those who had experienced one or more than one suicide.

We acknowledge the potential for non-response bias from those with positive experiences who did not feel motivated to record them, and also from those most distressed. The tendency for people bereaved by suicide to perceive self-stigma [16], blame, responsibility, and guilt [11] may have influenced respondents’ experiences of the press and also biased recall. Whilst objections to inaccurate or disrespectful reporting are generally understandable, some of the objections raised may have been based on unrealistic expectations that journalists should only report accounts favourable to the deceased or their friends and families. This might also reflect a response bias from those with most painful experiences. Where respondents castigated journalists for focusing on sensational details of the deceased, or for under-representing their accomplishments, their expectations may have been unfeasible. Where consultation was felt to be limited, this may have reflected journalists’ time pressures. It was also possible that frustration with journalists (and other agencies) represented a projection of anger about the death. The online survey allowed us to collect data from a large sample, but in not conducting interviews we lacked the opportunity to probe meaning where responses were ambiguous, and or to gain more in-depth information about attitudes and experiences. Reflexivity may have been limited by the primary coders being research psychiatrists, but the research team included a medical sociologist (FS). We also lacked the triangulation gained from examining linked journalistic material (press reports of the death; television coverage) or from gaining the perspectives of others in the network.

### Clinical and policy implications

Our finding that press behavior after a suicide can be distressing for the bereaved is concerning given their increased risk of suicide and psychiatric illness [11]. Negative press experiences risk re-traumatising vulnerable individuals by providing distressing reminders of the loss. Existing support guides [32] include a section on handling media attention and how to complain if this is intrusive or misrepresentative. However, this and other qualitative work [17, 31] suggests a need for more proactive support, such as bereavement support organisations providing a media spokesperson to act as an intermediary with the media. A spokesperson could help draft and release media statements featuring what details and/or photos the family felt comfortable revealing, accompanying them to safeguard privacy and rights throughout the interview process. This is likely to be acceptable to journalists in saving them time spent information-gathering and relieving them from the potential awkwardness of interviewing a distressed person. They would also be able to explain that although consultation with the journalist can sometimes be useful, there is no right of veto on what is eventually published, and that press freedom permits journalists to report any potentially shameful event if judged newsworthy, even where families would prefer they did not.

The divergent views we identified over acceptable levels of detail illustrate the tension between the twin purposes of media guidelines: to prevent further suicides, and to protect the bereaved. Whilst the majority of people we sampled reported negative experiences with the press, in some cases this was due to journalists adhering to media guidelines by avoiding mentioning the cause of death or being perceived not to have given it sufficient coverage. In other cases this was due to clear infringement of the codes of practice on privacy, harassment, and intrusion [8]. Qualitative work with journalists is needed to ascertain whether this is due to low awareness or indifference to such professional codes, or their lack of time to consult appropriately. Gaining a better understanding of these barriers, including research to understand journalists’ priorities and experiences in suicide-reporting and their attitudes to media guidelines, would help the policy community find ways of engaging with the media to address this. Some journalists express skepticism about the evidence for damaging effects of suicide reporting, perceiving guidelines as excessive restrictions on their freedom of speech [29] and a threat to the “duty to be truthful” [33]. They also struggle with the ambiguity in interpretation of the more nuanced recommendations [34]. Such frustrations are likely to be reinforced by the findings of this and previous research [17], portraying guidelines not only as excessively restrictive but also at odds with some relatives’ preferences. Such work should not be viewed as presenting a case for limiting press freedom, but as a means of helping journalists navigate suicide prevention recommendations more sensitively. The emotional proximity afforded through these perspectives from the bereaved could potentially temper reporting styles that this study’s participants found to be particularly upsetting. The experience of one 25 year old respondent highlights the value of taking the perspective of the bereaved: “*The press attended the inquest but were told to be sensitive and to imagine it was one of their family*”.

Our study also highlights the importance of consultation with the bereaved to address relatives’ preferences within the parameters of suicide prevention objectives. Media guidelines should be revised to suggest appropriate ways of initiating consultation, including how to approach relatives tactfully to explain plans for coverage and invite comments, providing them with an opportunity to be consulted sensitively on details reported, and explaining the rationale for them not having a final veto. When developing or revising guidelines, international experience suggests that involvement of journalists is critical [1, 35]. Future revisions of national media guidelines should start with a workshop involving key editors and journalists, to consider the evidence describing the effects of suicide reporting on relatives, and the wider evidence describing the population-level effects on suicidality. This would prompt discussion about how to harmonise the conflicting goals of journalistic freedom, suicide prevention, and minimising relatives’ distress. Once such guidelines have been agreed, they should be implemented using the endorsement of high-profile figures or organisations, assimilated into journalists’ vocational training, and supported with access to media advisory services [36]. International experience suggests that this relies on good relationships with newspaper editors [24, 25], and non-punitive approaches such as media awards to recognise responsible reporting [37]. Identification of an in-house journalist trained in sensitive suicide reporting might be an effective and acceptable means of providing guidance internally to colleagues. The proliferation of news reporting on social media, particularly Twitter [38], is a separate challenge likely to require self-regulation from within the online community.

### Future research

Whilst the current study describes the potential for subjective negative emotional effects of press reporting of suicide, it focuses on a majority white, female, young, and highly-educated sample. Further qualitative work is needed to explore the views of people in specific ethnic groups, older adults, and children. Mixed methods studies would also be of value, involving quantitative measures of psychiatric symptomatology (depression; post-traumatic stress disorder; suicidality), grief reactions, exposure to trauma reminders, and ratings of media coverage, as well as qualitative interviews. Qualitative work probing the views of news journalists in different countries would also help understand attitudes to local reporting guidelines, and guide the revision of existing guidelines. Positive experiences of co-production of media guidelines with journalists suggest that such work should be replicated on a country-by-country basis, involving people bereaved by suicide.

## Conclusions

We found that among a British sample of relatives and friends bereaved by suicide experiences were apparent of perceived press intrusion, invasion of privacy, and inaccurate reporting, including misleading representations of the deceased or the manner in which they died. These experiences were often negative, involving apparent transgressions of media guidelines on the reporting of suicide. However, in some cases, relatives’ objections related to instances of journalists following media guidelines. This distress caused to relatives through media coverage of a suicide is concerning given that it represents potential retraumatisaion of a group at theoretical risk of suicide. The personal perspectives provided by this work have the potential to be a powerful educational tool. They can help journalists in their approach to news reporting of suicide; balancing public interest with the preferences and sensitivities of the bereaved, and the wider aim of preventing further suicides. It would be helpful to amend existing media guidelines on the reporting of suicide to emphasise the importance of tactful consultation, accurate reporting, and respect for privacy in minimizing further distress to a vulnerable group.

## Data Availability

Quantitative and qualitative data collected for the UCL Bereavement Study are not publically available due to the risk of identifying participants but requests to analyse data should be made to the corresponding author, subject to internal peer review.

## Abbreviations

COREQ: Consolidated criteria for reporting qualitative research
HEI: Higher education institution
IQR: Inter-quartile range
SD: Standard deviation
UCL: University College London
UK: United Kingdom
WHO: World Health Organization
